# Carrageenan-Free Diet Shows Improved Glucose Tolerance and Insulin Signaling in Prediabetes: A Randomized, Pilot Clinical Trial

**DOI:** 10.1155/2020/8267980

**Published:** 2020-04-21

**Authors:** Leo Feferman, Sumit Bhattacharyya, Erin Oates, Nicole Haggerty, Tianxiu Wang, Krista Varady, Joanne K. Tobacman

**Affiliations:** ^1^Department of Medicine, College of Medicine, University of Illinois at Chicago and Jesse Brown VA Medical Center, Chicago, IL, USA; ^2^Department of Nutrition, College of Applied Health Sciences, University of Illinois at Chicago, Chicago, IL, USA; ^3^Department of Epidemiology and Biostatistics, College of Public Health, University of Illinois at Chicago, Chicago, IL, USA

## Abstract

**Objectives:**

Carrageenan is well known to cause inflammation and is used in laboratory experiments to study mediators and treatments of inflammation. However, carrageenan is added to hundreds of processed foods to improve texture. Previous work indicated that low concentrations of carrageenan in drinking water caused marked glucose intolerance and insulin resistance in a mouse model. This exploratory, clinical study tested the impact of the no-carrageenan diet in prediabetes. *Research Design and Methods*. Participants with prediabetes (*n* = 13), defined as HbA1c of 5.7%-6.4%, enrolled in a 12-week, randomized, parallel-arm, feeding trial. One group (*n* = 8) was provided all meals and snacks with no carrageenan. A second group (*n* = 5) received a similar diet with equivalent content of protein, fat, and carbohydrate, but with carrageenan. Blood samples were collected at baseline and during oral glucose tolerance tests at 6 and 12 weeks. The primary outcome measure was changed in %HbA1c between baseline and 12 weeks. Statistical analysis included paired and unpaired *t*-tests, correlations, and 2 × 2 ANOVAs.

**Results:**

Subjects on no carrageenan had declines in HbA1c and HOMA-IR (*p* = 0.006, *p* = 0.026; paired *t*-test, two tailed). They had increases in C-peptide (*p* = 0.029) and Matsuda Index (2.1 ± 0.7 to 4.8 ± 2.3; *p* = 0.052) and declines in serum IL-8, serum galectin-3, and neutrophil phospho-(Ser307/312)-IRS1 (*p* = 0.049, *p* = 0.003, and *p* = 0.006; paired *t*-tests, two tailed). Subjects on the diet with carrageenan had no significant changes in these parameters. Significant differences between no-carrageenan and carrageenan-containing diet groups for changes from baseline to 12 weeks occurred in C-peptide, phospho-Ser-IRS1, phospho-AKT1, and mononuclear cell arylsulfatase B (*p* = 0.007, *p* = 0.038, *p* = 0.0012, and *p* = 0.0008; 2 × 2 ANOVA). Significant correlations were evident between several of the variables.

**Conclusions:**

Findings indicate improvement in HbA1c and HOMA-IR in participants on no-carrageenan diets, but not in participants on carrageenan-containing diets. Significant differences between groups suggest that removing carrageenan may improve insulin signaling and glucose tolerance. Larger studies are needed to further consider the impact of carrageenan on development of diabetes.

## 1. Introduction

Carrageenan is added to hundreds of processed foods, due to its ability to improve the texture and the solubility of other ingredients in processed foods. Following extraction from red seaweed, carrageenan is obtained and incorporated into a wide variety of food products, as well as pharmaceuticals, cosmetics, and other manufactured items. Carrageenan is composed of sulfated or unsulfated D-galactose residues which are linked in alternating *β*-1,4 and *α*-1,3 galactose-galactose bonds. Three major types of carrageenan are used in food products. These are *ι* (iota), *κ* (kappa), and *λ* (lambda), which vary in the extent and sites of sulfation. Carrageenan is found in hundreds of food products, including ice cream, chocolate milk, yogurt, soymilk, beer, deli meats, infant formula, salad dressings, nutritional supplements, and many other processed foods [1, 2]. Daily intake of carrageenan in food was calculated to be about 100 mg/day in adults in the United States in the 1970s [1]. More recently, intake has been estimated by food industry publications to be 18-40 mg/kg/day, indicating potential intake of several grams daily [3]. In addition to its use in food products, carrageenan is added to a variety of other products, including toothpaste, room air deodorizers, and cosmetics. Coincidentally with widespread and increasing industrial uses, carrageenan has been used for decades in thousands of experiments in the scientific laboratory, due to its ability to provoke inflammation [1, 4]. Carrageenan-induced inflammation has been used to test the effectiveness of anti-inflammatory therapies and to investigate mediators of inflammation in experimental models, indicated by over 11,000 references in PubMed. Previous studies showed that carrageenan initiates inflammation in intestinal epithelial cells by activating a pathway of innate immunity mediated by TLR4-BCL10 and by production of reactive oxygen species (ROS) [5–9].

Since insulin resistance is associated with activation of TLR4-initiated inflammation [10–12], the impact of carrageenan exposure on glucose tolerance and insulin signaling in C57BL/6J mice was tested. When exposed to a low concentration of carrageenan in their water supply, the experimental mice were glucose intolerant by six days [13, 14]. Oral carrageenan caused systemic inflammation, leading to impaired insulin signaling in the mouse liver. Carrageenan exposure inhibited insulin signaling by effects on both hepatic phospho-insulin receptor substrate 1 (IRS1) and growth-factor receptor bound protein 10 (GRB10) [13–15]. Carrageenan increased phospho-(Ser307/312)-IRS1, a negative regulator of insulin signaling, and reduced phospho-Tyr-IRS1, a positive regulator of insulin signaling. These experiments showed that oral exposure to carrageenan produced extraintestinal inflammatory effects in rodents which led to glucose intolerance. Animal studies by food industry scientists have also demonstrated the impact of carrageenan exposure on glycosuria [16]. Additional mechanisms by which carrageenan can lead to insulin resistance have been reported by other investigators [17]. In addition, carrageenan increased serum galectin-3 and increased binding of galectin-3 with the insulin receptor in the carrageenan-induced mouse model of nonobese diabetes [18]. Since galectin-3 interaction with the insulin receptor has been identified as a mechanism of insulin resistance [19], these findings support an additional mechanism by which carrageenan exposure can impair insulin signaling.

In the typical Western diet, carrageenan is consumed in greater quantity than in the mice in our experiments. Average daily carrageenan consumption in the United States was estimated in one report to be 250 mg/day, or ~4.2 mg/kg (250 mg/60 kg) [20, 21], considerably less than the amount reported by the industry [3]. In our experiments in the C57BL/6J mouse model, carrageenan was supplied in the drinking water at a concentration of 10 *μ*g/ml. With an average consumption of about 5 ml of water per day in mice weighing about 25 g, daily carrageenan intake was ~2 mg/kg (=50 *μ*g/25 g), much less than the conservative estimate of daily human consumption [13].

This is the first study to directly address whether or not carrageenan intake in the human diet affects glucose tolerance and insulin resistance. We hypothesize that carrageenan-induced effects on insulin signaling and inflammation contribute to glucose intolerance and insulin resistance.

## 2. Research Design and Methods

### 2.1. Study Design

**Figure 1:**
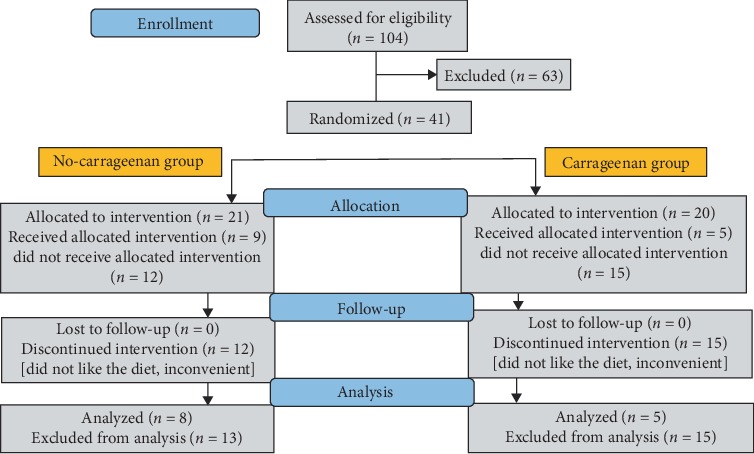
CONSORTdiagram. The CONSORT diagram shows the number of potential participants who responded to recruitment initiatives, the number who were excluded, the number who were randomized, the number who completed the study diets, and the number who completed laboratory procedures. One participant on review was found to have hemoglobin A1c below the cutoff for prediabetes and was excluded from analysis.

This pilot investigation was designed as a randomized, parallel-arm, clinical trial in which participants and study personnel who interacted with participants, with the exception of the study dietician, were blinded as to diet assignment. The study protocol was approved by the Institutional Review Board of the University of Illinois at Chicago (UIC), was supported by the American Diabetes Association, and was registered on the ClinicalTrials.gov website (“Impact of the no-Carrageenan Diet on Glucose Tolerance in Prediabetes” NCT02720393). The investigators had no conflict of interest with regard to the study. All participants provided written informed consent but were not asked to give consent for publication of individual data. Data supporting study conclusions are included within this report, and additional data are available upon request by written communication with JKT. CONSORT recommendations for transparent reporting of clinical trials were observed [22]. The study flow diagram is presented in Figure 1. Throughout the study, participants received their medical care from their personal physicians, who were blinded to the diet assignment. Data in all study records were anonymized, and records were compiled with a code for identification. Study procedures ended in June 2018.

The primary outcome measure was changed in HbA1c after 12 weeks of study participation. The secondary measure was changed in HOMA-IR, based on fasting glucose and insulin determinations at the time of glucose tolerance tests performed at onset of participation and after 12 weeks. Based on findings in previous mechanistic studies, additional outcome measures of interest were the inflammatory measures: serum IL-8, cellular phospho-(Ser307)-IRS1, and phospho-(Ser473)-AKT1. These had been shown to be modified in previous studies of carrageenan exposure [13–15].

### 2.2. Study Recruitment and Enrollment

Participants were recruited intermittently from January 2015 to February 2018. Potential participants responded to announcements posted in the General Medicine clinics of UI (University of Illinois) Health and in e-mails to the University of Illinois at Chicago (UIC) community. Respondents communicated by email or telephone to indicate their interest in participation, completed a telephone screening to assess eligibility, and scheduled a visit to the Clinical Research Center (CRC) on the UIC campus. Entry criteria included over 18 years of age, HbA1c of 5.7%-6.4% for at least 3 months, stable exercise routine, on no medication that affected blood sugar, able to pick up food weekly at the College of Applied Health Sciences (AHS) kitchen on the UIC campus, stable weight, and able to complete food questionnaires in English. Exclusion criteria included: previous diagnosis of diabetes; use of corticosteroids; serious underlying medical condition, including any disorder that affected red blood cell survival; food allergies or intolerances that impaired ability to adhere to study diet; or BMI ≥ 40.0 kg/m^2^. Participants agreed to not increase their exercise intensity or duration during the study.

### 2.3. Study Diet and Randomization

Participants were randomized by the study statistician to receive either the no-carrageenan diet or the comparable carrageenan-containing diet, including all meals and snacks for 12 weeks, using a computer-generated random allocation sequence. The CONSORT diagram (Figure 1) indicates the allocation of subjects: 21 to no-carrageenan diet group and 20 to carrageenan-containing diet group. Contents of the study diets were determined by study dieticians and study nutritionist-coinvestigator (KV), following conversations with food companies and evaluation of labels on commercial food products. Energy from dietary fat (~35-40% of energy), carbohydrate (~40-50% of energy), and protein (~15% of energy) at baseline and postintervention (Table 1) were similar for the no-carrageenan and the carrageenan-containing type of diet. Similar foods with or without carrageenan were selected for the majority of the dietary items. Caloric content was intended to maintain weight and baseline calorie needs were calculated by the Mifflin equation [23]. At baseline, subjects were classified as sedentary or lightly active based on self-reported activity level. The activity factor used for sedentary individuals was 1.2 and for lightly active individuals was 1.375.

Food was purchased and packaged in the metabolic kitchen at the University of Illinois, Chicago. Carrageenan-containing diets included 5 servings a day of carrageenan-containing foods (total estimated to be ~250 mg/day), predominantly in dairy food items and in processed deli meats. The amount of carrageenan consumed was assessed based on reported carrageenan content in the generic food products [24]. Participants completed daily logs indicating their adherence to consumption of the study diet contents and their consumption of nonstudy diet food items. These records were reviewed in detail by study investigators and compliance assessed by calculations of the number of nonstudy diet food items consumed and of the number of study diet items not eaten.

### 2.4. Study Procedures

At the initial visit, potential participants completed informed consent procedures and had a blood draw to confirm HbA1c level between 5.7% and 6.4%. Food log, campus map, and stool collection kit were distributed, and arrangements were made for food pickup at the College of AHS of UIC. Participants were provided the study diet for one week at a time, including three meals daily and snacks, in a wheeled, refrigerated container. Participants were weighed at the time of food pick up. Two-hour oral glucose tolerance tests (OGTT) were performed in the UIC CRC at 0, 6, and 12 weeks following a 12-hour overnight fast. Food intake was unrestricted prior to the initial OGTT. Glucose and insulin were measured in blood samples collected at baseline and at 30, 60, 90, and 120 minutes following administration of 75 g dextrose. C-peptide levels were measured in OGTT samples collected at 0- and 30-minute time points at 0 and 12 weeks (Supplementary Table 1). Participants also submitted stool samples which were tested for fecal calprotectin and microbiome. Quality of Life SF-36 questionnaires were completed, participants were weighed and measured, and vital signs were taken.

### 2.5. Laboratory Tests

Laboratory procedures were performed using standard techniques [5, 6, 13, 14]. Blood and stool samples were collected at 0 and 12 weeks, labeled by CRC designated, random, study identification, and assayed blindly to group assignment. Serum was separated and frozen for ELISA assays for IL-6 (DY206, R&D Systems, Bio-Techne, Minneapolis, MN), IL-8 (DY208, R&D), MCP-1 (DY279, R&D), insulin (DY8056, R&D), galectin-3 (DY1154, R&D), phospho-AKT(S473) (DYC-887B, R&D), phospho-IRS(Ser307) (#7287, Cell Signaling Technology, Danvers, MA), calprotectin 30-CALPHU-E01, ALPCO, Salem, NH), insulin (DY8056, R&D), and C-peptide (DICP00, R&D). Arylsulfatase B (ARSB, N-acetylgalactosamine-4-sulfatase) activity assay was performed as previously described using the exogenous substrate 4-methylumbelliferyl sulfate [25]. Human hemoglobin A1c assay kit (#80099, Crystal Chem, Elk Grove Village, IL) was used to measure HbA1c% in whole blood. Peripheral leukocytes were isolated from whole blood and separated into mononuclear and polymorphonuclear fractions by Polymorphprep™ (Axis-Shield, Inc., Norton, MA) [25–27]. Samples were frozen in DMSO, stored in a liquid nitrogen tank, and labeled by study identification number, without knowledge of participant diet assignment. Performance of some laboratory tests was limited by the volume of the samples that was available. All assays were performed using the manufacturer's recommended procedures, with technical replicates and known standards. HOMA values, Matsuda Index, oral disposition index, and QUICKI were calculated using glucose and insulin values from the OGTT (Supplementary Table 2) [28–32].

### 2.6. Statistics

Statistical analysis was performed to identify significant differences between pre- and postintervention test results in each study group (paired *t*-test, two tailed) and between the groups (2 × 2 ANOVA and unpaired *t*-tests, two tailed). Unpaired *t*-tests were used to compare the changes between baseline (0 weeks) and 12 weeks (postdietary intervention) by group. Correction was performed for unequal variance when the difference between the squares of the standard deviations of the group mean change was more than twofold. Pearson correlation coefficients *r* and associated  values were determined for the following: hemoglobin A1c, HOMA-IR, neutrophil phospho-(Ser307/312)-IRS1, and phospho-(Ser473)-AKT1, serum interleukin-8, monocyte arylsulfatase B, serum galectin-3, Matsuda Index, weight, and 0-30-minute difference in C-peptide and insulin values from the OGTTs. No previous data about the impact of carrageenan withdrawal on HbA1c% were available to inform the sample size estimate in this pilot study. High dropout rate was anticipated due to the stringent diet and 12-week duration. Initial randomization success was examined by testing group differences in preintervention data using *t*-tests. Weight and changes of weight were tested as potential confounding factors. All statistical tests were performed while controlling two-sided type I error probability of 0.05.

## 3. Results

### 3.1. Baseline Characteristics of Participants and Compliance with Study Diet

A total of 104 potential participants responded to an email notice to the UIC community about a study of carrageenan in prediabetes (Figure 1). Forty-one participants passed the screening and were randomized into carrageenan-containing (*n* = 20) and carrageenan-free diets (*n* = 21). The remaining 63 individuals were excluded due to a variety of reasons, including health issues, intolerance or allergy to the diet items, or not being able to commit sufficient time to the study. Twenty-seven participants dropped out after the randomization. Thirteen participants completed the study diets, including 8 participants on the no-carrageenan diet and 5 participants on the carrageenan-containing diet. Age range, race/ethnicity, and gender of participants were similar in the groups (Table 2).

Participants completed weekly logs about their consumption of the study diet which included all meals and snacks, and their intake of nonstudy food items. Diet compliance of participants who completed the study diets was similar in both groups. Average daily consumption of outside foods was 0.94 ± 0.62 food items. Average daily consumption of outside foods containing carrageenan was 0.15 ± 0.16 food items. The average consumption per participant of carrageenan-containing foods during the 12 weeks of the study was 9 in the no-carrageenan group (range: 0-24) and was 16 (range: 0-39) in the carrageenan-containing diet group. No harmful effects of the study diet were reported. Some participants noted that lactose intolerance was aggravated by the study diet, and this was managed by enzyme supplement and by consultation with the study dietician to reduce the number of dairy foods while maintaining the proportionate composition of protein, fat, and carbohydrate.

Weights were similar in both groups at baseline, and there was no significant difference in the change in weight between the groups. Both groups had declines in weight during the course of the study. Average decrease was 3.3 ± 4.2 kg for the carrageenan-containing diet group and 2.6 ± 3.1 kg for the carrageenan-free diet group.

### 3.2. Major Study Outcome Measures: Hemoglobin A1c % and HOMA-IR

Prediet and postdiet hemoglobin A1c (HbA1c) and homeostatic assessment of insulin resistance (HOMA-IR) results were compared between onset and at 12 weeks in participants on the carrageenan-containing study diet (Group 1; *n* = 5) and on the carrageenan-free study diet (Group 2; *n* = 8).

**Figure 2:**
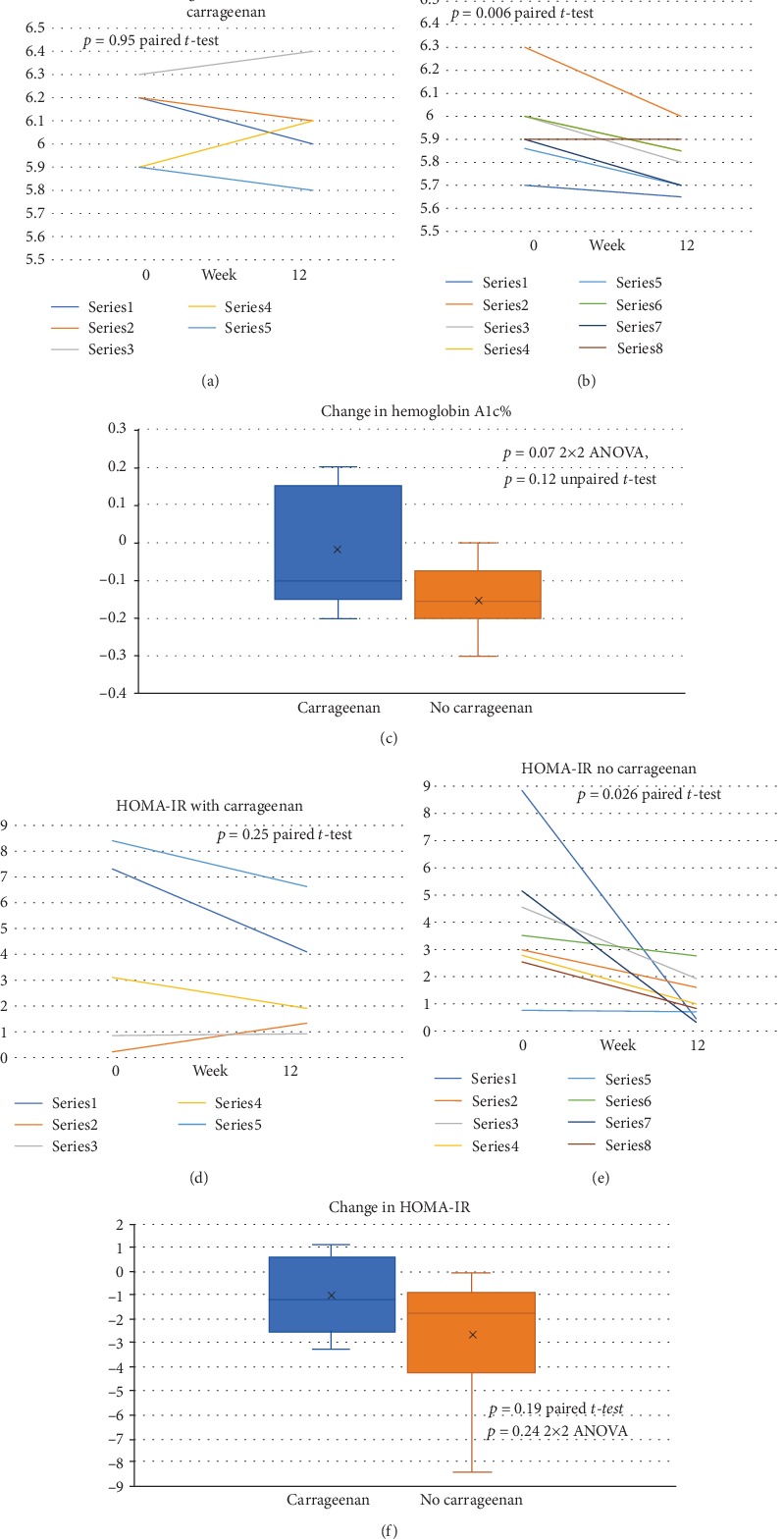
Major study outcome measures: hemoglobin A1c % and HOMA-IR. (a) Baseline (0 week) and 12-week values of hemoglobin (Hb) A1c (%) for the group on the carrageenan-containing diet are shown and were not significantly different (*p* = 0.95, paired *t*-test, two tailed, *n* = 5). (b) The difference between the 0-week and 12-week HbA1c values were significantly different for participants on the no-carrageenan diet (*p* = 0.006; paired *t*-test, two tailed, *n* = 8). (c) The distribution of changes between the two groups was not significant (*p* = 0.12, unpaired *t*-test, two tailed, unequal variance; *p* = 0.068, 2 × 2 ANOVA). (d) The differences between baseline and 12-week HOMA-IR scores were not significantly different for individuals on the carrageenan-containing diet (*p* = 0.25, paired *t*-test, two tailed, *n* = 5). (e) The differences between the baseline and 12-week HOMA-IR values were significantly different for the individuals on the no-carrageenan diet (*p* = 0.026, paired *t*-test, two tailed, unequal variance, *n* = 8). F. The changes in HOMA-IR scores between the no-carrageenan and carrageenan-containing groups were not significantly different.

Baseline hemoglobin (Hb) A1c values were similar in the groups. Average HbA1c for the carrageenan-free diet group declined significantly (*p* = 0.006, paired *t*-test, and two tailed), whereas the value for the carrageenan-containing diet group was unchanged (*p* = 0.95) (Figures 2(a) and 2(b)). The difference between the groups was not significant (*p* = 0.12, unpaired *t*-test, two tailed, and unequal variance) (Figure 2(c)).

The baseline values for HOMA-IR had no significant difference between the groups. The average decline for the carrageenan-containing controlled diet group was 1.00 ± 1.67 (*p* = 0.25, paired *t*-test, and two tailed) (Figure 2(d)). In contrast, the HOMA-IR of the carrageenan-free diet group declined by 2.69 ± 2.71 (*p* = 0.026, paired *t*-test, and two-tailed) (Figure 2(e)). The difference between the two groups was not significant (Figure 2(f)).

### 3.3. Insulin, C-Peptide, and Matsuda Index from Oral Glucose Tolerance Tests by Group at 0 and 12 Weeks

**Figure 3:**
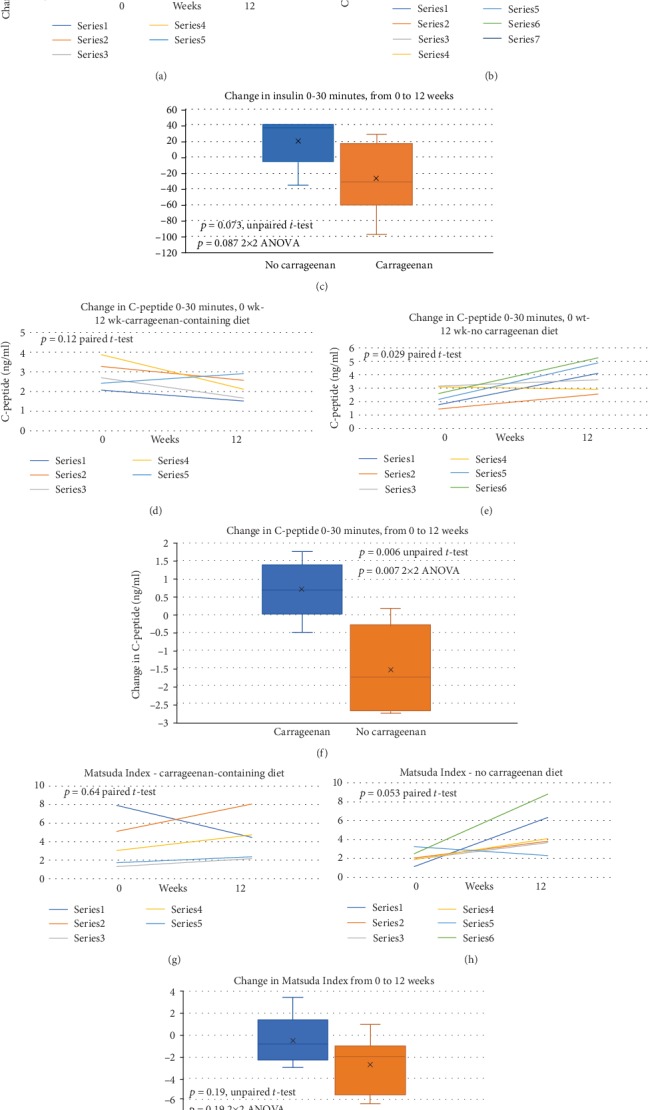
Impact of diet on changes in 0-30 minute insulin and C-peptide levels in oral glucose tolerance tests and on changes in Matsuda Index between 0 and 12 weeks. (a). Serum Insulin was measured at baseline and at 30, 60, 90, and 120 minutes following administration of oral glucose load in glucose tolerance tests. The differences between 0 and 30 minutes were determined, and these levels at baseline and after dietary intervention for 12 weeks are shown. No difference was evident for individuals on the carrageenan-containing diet (paired *t*-test, two tailed, and *n* = 5). (b). For individuals on the no-carrageenan diet, there was no significant difference between the baseline and 12-week levels (*n* = 7). (c). No significant differences between the no-carrageenan and carrageenan-containing diet groups were detected (*p* = 0.073, unpaired *t*-test, two tailed, and unequal variance; *p* = 0.087, 2 × 2 ANOVA) for the change from 0 to 12 weeks in the difference between 0 and 30 minutes. (d). Serum C-peptide levels were measured at baseline and at 30 minutes following administration of oral glucose in the OGTT. The differences between the 0 and 30 minutes values were compared between baseline and 12 weeks. No difference was detected in the individuals on the carrageenan-containing diet (*p* = 0.12, paired *t*-test, two tailed, and *n* = 5). (e). For participants on the no-carrageenan diet, there was a significant difference between the baseline and the 12-week levels (*p* = 0.029, paired *t*-test, two tailed, and *n* = 6). (f). The differences between 0 and 30 minutes were compared and were significant (*p* = 0.006, unpaired *t*-test, two tailed, and unequal variance; *p* = 0.007, 2 × 2 ANOVA) between the change from 0 to 12 weeks in the no-carrageenan vs. the carrageenan-diet group. (g). Matsuda Index was calculated at baseline and 12 weeks using the values of insulin and glucose from the OGTT. No significant difference was detected between baseline and 12 weeks for subjects on the carrageenan-containing diet (*n* = 5). (h). The difference between individuals on the no-carrageenan diet approached statistical significance (*p* = 0.053, paired *t*-test, two tailed, *n* = 6). (i). No significant difference was evident between the groups.

Values for the change between 0 and 30 minute values of insulin (mIU/L) obtained during oral glucose tolerance tests (OGTT) at 0 and 12 weeks are shown (Figures 3(a) and 3(b)). Paired *t*-tests indicated significant differences in participants on the no-carrageenan diet, but not on the carrageenan-containing diet. The distribution of the changes in the groups between 0 and 12 weeks is presented (Figure 3(c)). No significant differences were demonstrated between the groups (unpaired *t*-test and 2 × 2 ANOVA).

Changes in C-peptide values between 0 and 30 minutes were compared between baseline and 12 weeks in each group. Paired *t*-tests showed significant increases in the C-peptide values (*p* = 0.029) in the no-carrageenan diet group, but not in the carrageenan-containing diet group (*p* = 0.123) (Figures 3(d) and 3(e)). The difference between the two groups was significant (Figure 3(f)) (*p* = 0.006, unpaired *t*-test, two tailed, and unequal variance; *p* = 0.0072, 2 × 2 ANOVA).

Matsuda Index was calculated using the results of the OGTT. Differences between baseline and 12-week results were calculated for each group by paired *t*-tests (Figures 3(g) and 3(h)) (*p* = 0.64 with carrageenan and *p* = 0.052 in the no-carrageenan group). In the no-carrageenan diet group, mean Matsuda Index increased from 2.1 ± 0.7 to 4.8 ± 2.3 and in the carrageenan-diet group, Matsuda Index increased from 3.8 ± 2.7 to 4.4 ± 2.4. Differences in the groups were not significant by unpaired *t*-test and 2 × 2 ANOVA (Figure 3(i)).

### 3.4. Impact of Diet on Inflammation: Effects on Serum Interleukin-8 and Fecal Calprotectin

**Figure 4:**
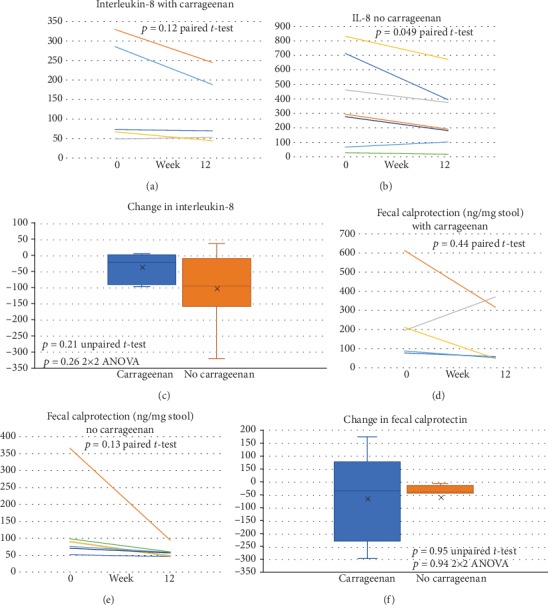
Impact of diet on inflammation: effects on serum interleukin-8 and fecal calprotectin. (a). Baseline and 12-week levels of serum IL-8 levels (pg/ml) were not significantly different in participants on the control diet (*n* = 5). (b). In contrast, the change in IL-8 levels between 0 and 12 weeks was significant for the participants on the no-carrageenan diet (*p* = 0.049; paired *t*-test, two tailed, unequal variance, and *n* = 7). (c). The changes in IL-8 postdietary intervention were not significantly different between the groups. (d). Fecal calprotectin was not significantly changed between 0 and 12 weeks in participants on the diet with carrageenan (*n* = 5). (e). The difference between baseline and study end was not significantly different for participants on the no-carrageenan diet (*p* = 0.13; paired *t*-test, two tailed, *n* = 7). (f). No difference between groups was detected.

The distribution of the initial values for the inflammatory parameters was similar for the two groups. The average result for IL-8 in the carrageenan-containing diet group showed no significant change (*p* = 0.12, paired *t*-test, two tailed, *n* = 5) (Figure 4(a)). In contrast, the carrageenan-free diet group had a significant decline (*p* = 0.049, paired *t*-test, two tailed, *n* = 8) (Figure 4(b)). The difference in the changes in values for the two groups was not significant (Figure 4(c)). Measurements of fecal calprotectin (Figures 4(d)–4(f)), IL-6, and MCP-1 (not shown) were not significantly different between baseline and final values in either group or between groups.

### 3.5. Impact of Diet on Cell-Based Inflammatory Parameters; Effects on Neutrophil Phospho-(Ser307/312)-IRS1 and Phospho-(Ser473)-AKT1

**Figure 5:**
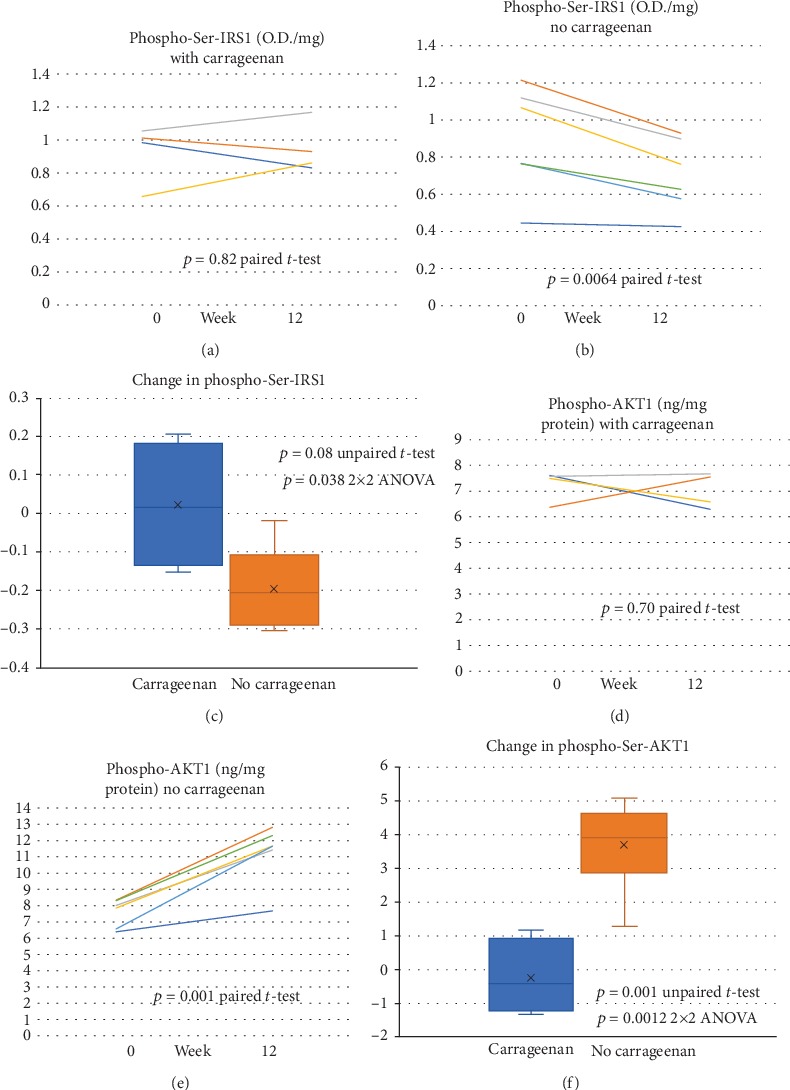
Impact of diet on cell-based inflammatory parameters; effects on neutrophil phospho-(Ser307/312)-IRS1 and phospho-(Ser473)-AKT1. (a). Baseline and 12-week values of neutrophil phospho-(Ser307/312)-IRS1, measured as optical density (O.D.)/mg cell protein, were similar for the carrageenan-containing diet group (*n* = 4). (b). In contrast, the 0- to 12-week decline was significant in the no-carrageenan group (*p* = 0.006, paired t-test, two tailed, and *n* = 6). (c). The changes in neutrophil phospho-(Ser307/312)-IRS1 between the no-carrageenan and carrageenan-containing diet groups were not significantly different (*p* = 0.08, unpaired *t*-test, two tailed, and equal variance; *p* = 0.00122 × 2 ANOVA). (d). Phospho-(Ser473)-AKT1 (ng/mg protein) was measured by ELISA in neutrophils from participants. There was no significant difference between the baseline and 12-week results in subjects on the carrageenan-containing study diet (*p* = 0.70, paired *t*-test, two tailed, *n* = 4). (e). In contrast, participants on the no-carrageenan diet had marked increase in phospho-(Ser473)-AKT1, consistent with the decline in phospho-(Ser307/312)-IRS1 (*p* = 0.001, paired *t*-test, two tailed, and *n* = 6). (f). There was a significant difference between carrageenan-containing and no-carrageenan diet groups (*p* = 0.0012, unpaired *t*-test, two tailed, and equal variance).

Phospho-(Ser307/312)-IRS1, a marker of inhibition of insulin signaling at the intersection of inflammatory and insulin signaling pathways, was compared in neutrophils from the participants. The distribution of the initial values was similar in the two groups. The carrageenan-containing diet group showed no significant change between onset and final values (*p* = 0.82, paired *t*-test, and *n* = 4) **(**Figure 5(a)). In contrast, the no-carrageenan group had a significant decline (*p* = 0.006, paired *t*-test, two tailed, and *n* = 6) (Figure 5(b)). The difference between the groups was not significant (*p* = 0.08, unpaired *t*-test, two tailed, and unequal variance) (Figure 5(c)).

Neutrophil phospho-(Ser473)-AKT1,2 increased in all individuals tested on the no-carrageenan diet (*p* = 0.001, paired *t*-test, two tailed, and *n* = 6) following 12 weeks of intervention. The carrageenan-containing diet group had no significant change by paired *t*-test (*p* = 0.70, *n* = 4) (Figures 5(d) and 5(e)). Baseline values were similar between the no-carrageenan and carrageenan-containing diet groups. The changes between the groups were significant (*p* = 0.001, unpaired *t*-test, two tailed, and equal variance; *p* = 0.0012 2 × 2 ANOVA) (Figure 5(f)).

### 3.6. Impact of Diet on Mononuclear Arylsulfatase B and Serum Galectin-3

**Figure 6:**
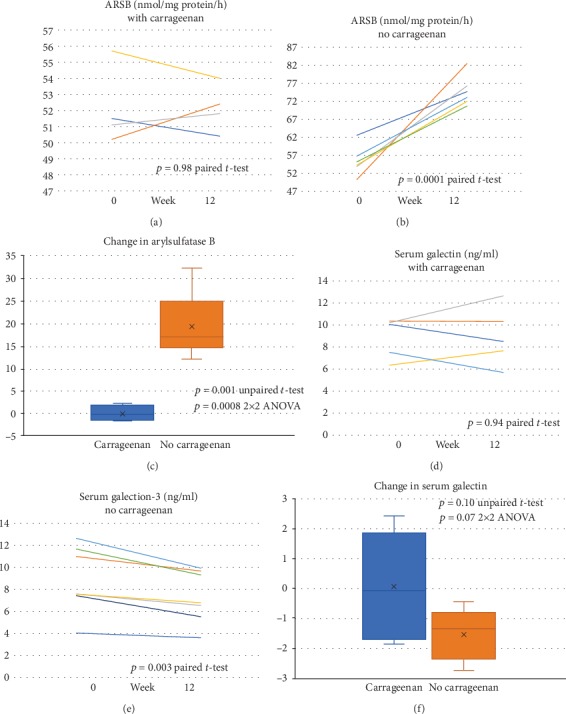
Impact of diet on mononuclear arylsulfatase B and serum galectin-3. (a). Mononuclear ARSB activity (nmol/mg protein/h) was not significantly different between baseline and 12 weeks in the participants on the carrageenan-containing diet (*p* = 0.98, paired *t*- test, two tailed, and *n* = 4). (b). In the no-carrageenan diet, the mononuclear ARSB activity increased significantly (*p* = 0.001, paired *t*-test, two tailed, and *n* = 6). (c). The changes in mononuclear ARSB activity were different between the carrageenan-free and carrageenan-containing diet groups (*p* = 0.0008, unpaired *t*-test, two tailed, and unequal variance). (d). Serum galectin-3 was similar at 0 and 12 weeks in the carrageenan-containing diet group (*p* = 0.94, paired *t*-test, two tailed, *n* = 4). (e). In the carrageenan-free diet group, serum galectin-3 declined significantly between baseline and 12 weeks (*p* = 0.003, paired *t*-test, two tailed, *n* = 6). (f). The difference in the change in serum galectin-3 between the two groups was determined (*p* = 0.13, unpaired *t*-test, two tailed, and unequal variance).

Arylsulfatase B (ARSB; N-acetylgalactosamine-4-sulfatase) activity was measured in circulating mononuclear cells, since it was previously shown to decline when colonic epithelial cells were treated with carrageenan and may be useful as a surrogate marker for carrageenan exposure [33]. Distribution of the initial values was similar (Figures 6(a) and 6(b)). Following the no-carrageenan diet, all ARSB activity values increased. Average values increased significantly, from 55.5 ± 4.1 nmol/mg protein/h to 74.9 ± 4.2 nmol/mg protein/h (*p* = 0.001, paired *t*-test, two tailed, and *n* = 6). The average result for the carrageenan-containing diet group was 52.1 ± 2.4 nmol/mg protein/h at baseline and 52.2 ± 1.5 nmol/mg protein/h at 12 weeks, showing no significant change (*p* = 0.98, paired *t*-test, and *n* = 4). The difference in the changes between the two groups was significant (*p* = 0.0008, unpaired *t*-test, two tailed, and unequal variance; *p* = 0.0008, 2×2 ANOVA) (Figure 6(c)).

Decline in ARSB leads to increased chondroitin 4-sulfate (C4S), since ARSB is required for the degradation of C4S. Previously, galectin-3 was shown to bind less to chondroitin 4-sulfate when ARSB was reduced [34], and to bind with the insulin receptor and contribute to insulin resistance [19]. Hence, increased ARSB activity may lead to a decline in serum galectin-3 and to reduced insulin resistance. To test this potential mechanism by which carrageenan can contribute to insulin resistance, serum galectin-3 levels were measured in study participants. Galectin-3 values were similar at study onset and declined significantly in the no-carrageenan diet group, from 8.56 ± 2.93 ng/ml to 7.25 ± 2.23 ng/ml (*p* = 0.003, paired *t*-test, two tailed, and *n* = 8) (Figure 6(e)), with no change in the carrageenan-containing diet group (*p* = 0.94, paired *t*-test, and *n* = 5) (Figure 6(d)). The overall difference between carrageenan-free and carrageenan-containing diets was not significant (Figure 6(f)).

### 3.7. Correlations between Variables of Interest

Correlations between variables for which paired *t*-tests or 2 × 2 ANOVA were significant were determined (Table 3). The most significant positive correlations were between the changes between baseline and 12 weeks in: HbA1c and p-Ser-IRS1 (*r* = 0.94, *p* < 0.0001); galectin-3 and p-Ser-IRS1 (*r* = 0.89, *p* = 0.0006); C-peptide and insulin values, using the differences between 0 and 30 minutes from the OGTTs (*r* = 0.88, *p* = 0.0004); and arylsulfatase B and phospho-AKT1 (*r* = 0.88, *p* = 0.0009). Other significant results, including the negative correlation between phospho-Ser-IRS1 and phospho-Ser-AKT (*r* = −0.72, *p* = 0.018), are consistent with established insulin signaling pathways.

## 4. Discussion

Study results indicate that participants on the no-carrageenan study diet had declines in HbA1c and HOMA-IR (paired *t*-tests). In contrast, the participants who received the carrageenan-containing study diet did not have any significant declines in HbA1c or HOMA-IR. Other significant before-after changes in the participants on the no-carrageenan diet include declines in serum IL-8, serum galectin-3, and neutrophil phospho-(Serine307/312)-IRS1. Increases include arylsulfatase B, phospho-(Ser473)-AKT1, C-peptide, and Matsuda Index. Significant differences between groups occurred in neutrophil phospho-(Ser473)-AKT and neutrophil phospho-(Ser307/312)-IRS1, C-peptide, and mononuclear ARSB activities. These results suggest that the decline in carrageenan exposure led to improved insulin signaling. Although study numbers are small, the data show significant improvement in neutrophil mediators of insulin signaling in participants on the no-carrageenan diet, with increased phospho-(Ser473)-AKT1 and reduced phospho-(Ser307/312)-IRS1. These findings are consistent with our previously published findings in hepatic tissue of carrageenan-exposed mice, in which carrageenan exposure increased phospho-(Ser307/312)-IRS1 and reduced phospho-(Ser473)-AKT1, and thereby inhibited insulin signaling and impaired glucose tolerance [13, 14, 18]. The unexpected increase in C-peptide in the participants on the carrageenan-free diet suggests that removal of carrageenan may also lead to increased insulin production or secretion.

Although the study diets were not intended for to weight loss, mean weight declined in both groups. There was no significant difference in the weight change between the groups, and average weight loss was less in the no-carrageenan diet group. Although it is unclear why subjects lost weight, the participants may have found the three-day rotating menu to be unappealing for 12 weeks. They may have underreported their lack of consumption of the study diet, since food allotment was adjusted to provide enough calories to maintain weight. To our knowledge, participants did not increase their physical activity during study participation. A decline in HbA1c did not occur in the carrageenan-exposed group, although average weight loss was greater than in the no-carrageenan group. In our previously reported mouse studies, carrageenan exposure, in contrast to high-fat diet, was not associated with weight gain and the carrageenan model of diabetes is a nonobese model [14, 18]. Additional studies are needed to determine how carrageenan exposure or removal of carrageenan might affect obesity-associated inflammation in individuals with diabetes or prediabetes.

The implications of the study findings are limited by the small sample size, since a type 1 error cannot be excluded. Initial recruitment was high, but retention of recruited study participants was less than anticipated. Participants indicated dissatisfaction with being on the restricted study diet for 12 weeks and with the inconvenience of picking up their food weekly. Abstinence from beer, which generally contains carrageenan, was a major factor in the reluctance of some individuals to follow the study diet. Other study limitations included the relatively short duration of the study and the occurrence of weight loss in participants, who likely did not eat all of the food provided in the study diets due to their personal preferences. Also, most study participants were female, so findings may be related to gender. No specific biomarker of carrageenan consumption is available, limiting confirmation of dietary compliance/noncompliance, and no lipid data are available for study participants. Another study limitation is the lack of specific information about volume and intensity of exercise throughout the study. Although a criterion of participation was agreed to not modify exercise during the study, it is possible that change in exercise contributed to weight loss and improved glucose tolerance.

Study findings support and build upon our previous findings with carrageenan-fed mice and carrageenan-treated human cells, which linked carrageenan exposure with glucose intolerance and insulin resistance. Previous findings elucidated the pathways by which carrageenan interferes with the insulin response, by effects on signaling cascades mediated by ROS, phospho-IRS1, and GRB10. Now, we include effects of carrageenan on ARSB and galectin-3 as additional mechanisms which may contribute to impaired insulin signaling. Identification of the impact of carrageenan withdrawal on mononuclear arylsulfatase B activity and on serum galectin-3 is biochemical evidence that ingestion of carrageenan can affect the metabolism of sulfated glycosaminoglycans (GAGs) and the molecular pathways regulated by more or less binding with the sulfated GAGs. The observed decline in serum galectin-3 is consistent with previous reports of mimicry by carrageenan of the naturally occurring sulfated GAGs, particularly chondroitin 4-sulfate (C4S) [35]. Carrageenan was shown to reduce activity of ARSB, and the binding of galectin-3 to C4S was reported to decline when ARSB activity was less and C4S was thereby increased [34]. Hence, carrageenan exposure may also impact insulin signaling through ARSB and chondroitin 4-sulfate-mediated changes in free galectin-3, as suggested by the findings in this report and animal studies [18]. Li et al. reported that hematopoietic-derived galectin-3 binds to the insulin receptor and inhibits downstream signaling [19]. We suggest that carrageenan-induced changes in ARSB and C4S may impact galectin-3 availability and contribute to the effect on HbA1c that we report. Also, in other works, a decline in ARSB led to increased C4S and increased binding of C4S with SHP2 (PTPN11), the ubiquitous nonreceptor tyrosine phosphatase [36–38]. Increased binding of SHP2 with C4S reduced SHP2 activity, leading to sustained phosphorylation of several mediators, including phospho-ERK1/2, phospho-JNK, and phospho-p38 MAPK. Inhibition of SHP2 has been linked to changes in insulin signaling [39], and it is possible that effects of carrageenan, mediated through ARSB and chondroitin sulfation, may modify SHP2 activity and impact insulin signaling through these pathways.

Different types of dietary interventions have been previously evaluated for effectiveness in prevention of T2D [40–45]. The effects of these interventions have often been attributed to effects on beta cells, although effects on inflammation have also been considered. The impact of carrageenan on beta cells is unknown, and the increase in C-peptide in this study is a new finding. Maintenance of weight loss and sustained long-term dietary modification beyond the duration of study participation are well-recognized challenges in diabetes prevention through dietary intervention [45].

This report suggests that in individuals with prediabetes removal from the diet of the common food additive carrageenan is a novel intervention that may help prevent T2D. The study diet completely eliminated the proinflammatory, commonly used food additive carrageenan from the diet. In twelve weeks, HbA1c declined by an average of 0.12% in study participants on the no-carrageenan diet. It is uncertain whether or not the HbA1c would continue to decline with ongoing removal of carrageenan from the diet. Further studies are needed to clarify the precise mechanisms by which the specific elimination of carrageenan-containing foods can affect glucose tolerance in the long term. Although food industry scientists have published several studies in support of the safety of carrageenan [3, 46], the overwhelming evidence in the literature and that reported on PubMed indicates that carrageenan exposure predictably causes inflammation and leads to significant physiological consequences [47]. The impact of removing carrageenan from the diet in patients with established diabetes and impaired beta cell function needs further investigation. Effects of carrageenan on the fecal microbiome, lymphocyte subsets, lipid parameters, and other inflammatory parameters may also contribute to the impact of carrageenan on glucose metabolism and human health.

## Figures and Tables

**Table 1:** Baseline and postintervention food intake by group.

Nutrient	Carrageenan-diet group (*n* = 5)		No-carrageenan diet group (*n* = 8)	
	Baseline	Postintervention	Baseline	Postintervention
Energy (kcal/d)	2425	2405	2250	2211
Protein (% of energy)	16	15	14	15
Carbohydrates (% of energy)	42	48	46	49
Fat (% of energy)	42	37	40	36
Fiber (g)	21	22	20	19
Cholesterol (mg)	301	285	336	296

**Table 2:** Baseline characteristics of study participants.

Characteristic	Carrageenan-diet group (*n* = 5)	No- carrageenan diet group (*n* = 8)
Male : Female	1 : 4	1 : 7
Age (years)	41.6 ± 14.9	50.4 ± 9.9
Age range (years)	22-63	31-64
Race/ethnicity		
Asian	1	1
African-American	3	6
Caucasian	1	0
Hispanic or Latino	0	1
Not Hispanic or Latino	5	7
Body weight (kg)	103 ± 22	87 ± 19
BMI (kg/m^2^)	34.5 ± 5.3	31.2 ± 5.5
Baseline hemoglobin A1c (%)	6.09 ± 0.19	5.94 ± 0.17
Baseline fasting glucose (mg/dl)	96.2 ± 19.5	94.5 ± 11.6
Baseline 2-hour glucose in OGTT (mg/dl)	139.3 ± 75.1	164.4 ± 53.0
Baseline fasting insulin (*μ*IU/ml)	17.7 ± 17.5	16.6 ± 9.8
Baseline 2-hr insulin in OGTT (*μ*IU/ml)	69.1 ± 49.7	86.8 ± 46.7
Baseline C-peptide (ng/ml)	0.86 ± 0.61	1.06 ± 0.51
Baseline Matsuda Index	3.83 ± 2.73	2.12 ± 0.71
Baseline serum galectin-3 (ng/ml)	8.90 ± 1.84	8.86 ± 3.03
Baseline arylsulfatase B (nmol/mg protein/h)	52.1 ± 2.44	55.5 ± 4.08

All values are reported as mean ± SD (standard deviation).

**Table 3:** Pearson's correlation coefficient (*r*) for changes from baseline to 12 weeks between variables^∗^.

	Hemoglobin A1c	HOMA-IR	Phospho-serine-IRS1	Interleukin-8	Arylsulfatase B	Galectin-3	Phospho-serine-AKT	Insulin#	C-peptide^‡^	Matsuda Index	Weight
Hemoglobin A1c	1	-0.11	*0.94*	0.12	**-0.73**	*0.75*	**-0.66**	-0.33	-0.54	-0.21	0.30
		0.72	<0.0001	0.71	0.012	0.005	0.039	0.30	0.08	0.54	0.32
	13	13	10	12	10	12	10	12	11	11	13

HOMA-IR		1	-0.33	*0.76*	-0.14	-0.20	-0.027	-0.36	-0.52	-0.40	-0.01
			0.36	0.004	0.70	0.54	0.94	0.25	0.10	0.22	0.47
		13	10	12	10	12	10	12	11	11	13

Phospho-serine-IRS1			1	-0.07	**-0.75**	*0.89*	**-0.72**	-0.46	-0.58	-0.03	0.10
		0.85	0.01	0.0006	0.018	0.18	0.10	0.95	0.79
	10	10	10	10	10	10	9	9	10

Interleukin-8				1	-0.28	-0.02	-0.11	-0.12	-0.43	-0.39	-0.09
		0.44	0.94	0.75	0.71	0.18	0.24	0.78
	12	10	12	10	12	11	11	12

Arylsulfatase B					1	-0.55	*0.88*	0.36	0.60	0.39	-0.21
		0.09	0.0009	0.31	0.08	0.30	0.56
	10	10	10	10	9	9	10

Galectin-3						1	-0.59	**-0.68**	**-0.68**	-0.043	-0.026
		0.07	0.016	0.020	0.90	0.94
	12	10	12	11	11	12

Phospho-serine-AKT							1	0.42	0.53	0.57	0.12
		0.22	0.14	0.11	0.75
	10	10	9	9	10

Insulin								1	*0.88*	0.27	0.067
		0.0004	0.52	0.84
	12	11	10	12

C-peptide									1	0.23	-0.063
		0.52	0.85
	11	10	11

Matsuda Index										1	0.25
		0.47
	11	11

Weight											1

	13

^∗^Data include Pearson's correlation coefficient *r* (first line), *p* value (second line), and number of subjects (carrageenan and no-carrageenan diet combined; third line) for whom correlation was calculated. *R* values highlighted in **italic** are significant positive correlations, and *r* values highlighted in **bold** are significant negative correlations. ^#^ refers to the difference in the change in insulin levels between 0 and 30 minutes which occurred between 0 and 12 weeks. ^‡^ refers to the difference in the change in C-peptide levels between 0 and 30 minutes, which occurred between 0 and 12 weeks.

## Data Availability

Data supporting study conclusions are included within this report, and additional data are available from the corresponding author upon request.

## Supplementary Materials

Supplementary Materials Supplementary Table 1: “Results of glucose, insulin, and C-peptide determinations during OGTT at baseline and postintervention.” Supplementary Table 2: “Oral disposition index, HOMA-IR, Matsuda Index, QUICKI, and %S at 0 and 12 weeks.”
