# Supplementary material for: Carrageenan-Free Diet Shows Improved Glucose Tolerance and Insulin Signaling in Prediabetes: A Randomized, Pilot Clinical Trial
Source: J Diabetes Res. 2020 Apr 21;2020:8267980. doi: 10.1155/2020/8267980 (PMC7191375; doi:10.1155/2020/8267980)
Supplement: Supplementary Materials — Supplementary Table 1: “Results of glucose, insulin, and C-peptide determinations during OGTT at baseline and postintervention.” Supplementary Table 2: “Oral disposition index, HOMA-IR, Matsuda Index, QUICKI, and %S at 0 and 12 weeks.” [file 8267980.f1.pdf]

Supplementary Table 1. Results of glucose, insulin, and C-peptide determinations during OGTT at baseline and post-intervention.

**DIET**		**Baseline OGTT**						**12-week OGTT**				
**No-carrageenan**	***Minutes***	***0***	***30***	***60***	***90***	***120***		***0***	***30***	***60***	***90***	***120***
1 [mg/dl]	Glucose	105	164	209	207	179	Glucose	90	149	196	207	172
[µIU/ml]	Insulin	34.1	72.6	188.4	130.1	141.2	Insulin	2.0	75.8	88	115.9	79.1
[ng/ml]	C-peptide	1.93	3.69				C-peptide	1.81	5.93			
2	Glucose	109	170	190	230	218	Glucose	93	156	222	230	197
	Insulin	11.1	35.4	86.7	181.7	213.5	Insulin	7.0	14.6	58.2	67.4	159.8
	C-peptide	0.61	2.05				C-peptide	0.61	3.17			
3	Glucose	87	130	163	149	146	Glucose	99	137	126	132	129
	Insulin	21.2	83.3	130.3	142	130.9	Insulin	7.9	55.7	74.6	98.1	130.9
	C-peptide	1.25	4.41				C-peptide	1.28	4.91			
4	Glucose	115	216	273	303	309	Glucose	120	187	264	288	275
	Insulin	9.8	28.5	128.4	149.0	120.5	Insulin	3.4	23.8	82.1	83.6	120.7
	C-peptide	0.56	3.66				C-peptide	0.51	3.43			
5	Glucose	78	106	133	130	130	Glucose	85	144	172	161	139
	Insulin	4.0	7.6	-	9.1	20.5	Insulin	3.4	52.0	95.9	64.7	53.3
	C-peptide						C-peptide					
6	Glucose	89	177	160	147	124	Glucose	95	150	182	160	112
	Insulin	16.0	65.6	44.0	46.6	32.4	Insulin	11.8	121.4	171.3	117.4	78.1
	C-peptide	0.83	2.99				C-peptide	0.62	5.52			
7	Glucose	104	137	160	159	138	Glucose	89	129	169	185	124
	Insulin	20.1	65.8	60.6	54.4	43.4	Insulin	1.5	144.3	51.9	56.8	26.5
	C-peptide	1.18	3.79				C-peptide	0.58	5.85			
8	Glucose	90	139	155	175	141	Glucose	85	134	173	187	167
	Insulin	11.4	33.3				Insulin	4.0	60.0			
	C-peptide						C-peptide					

**DIET**	**Baseline OGTT**						**12-week OGTT**					
**Carrageenan-containing diet**	**Minutes**	**0**	**30**	**60**	**90**	**120**	**Minutes**	**0**	**30**	**60**	**90**	**120**
1	Glucose	90	163	216	213	170	Glucose	86	146	185	189	174
	Insulin	1	46.4	101.2	185.1	117	Insulin	6.3	29.3	74.4	71.2	100.9
	C-peptide	0.25	2.32				C-peptide	0.25	1.76			
2	Glucose	91	173	174	74	61	Glucose	85	174	166	104	75
	Insulin	3.8	84.8	153.7	78.7	71.6	Insulin	4.4	45.3	72.0	5.1	1.2
	C-peptide	0.38	3.66				C-peptide	0.32	2.89			
3	Glucose	105	175	206	228	234	Glucose	126	173	203	234	250
	Insulin	28.2	84.4	110.3	136.0	123.4	Insulin	13.2	28.8	65.2	91.9	128.7
	C-peptide	1.16	3.85				C-peptide	1.14	2.80			
4	Glucose	96	133	118	112	114	Glucose	106	134	155	124	126
	Insulin	13.1	88.0	81.9	87.6	57.1	Insulin	7.2	46.1	69.9	33.4	42.7
	C-peptide	0.76	4.64				C-peptide	0.79	2.91			
5	Glucose	80	126	132	114	105	Glucose	78	97	80	83	71
	Insulin	42.5	106.2	96.4	62.5	103.9	Insulin	34.4	133.6	42.3	88.2	71.9
	C-peptide	1.74	4.16				C-peptide	1.77	4.68			

Supplementary Table 2. Oral disposition index, HOMA-IR, Matsuda Index, QUICKI, and %S at 0 and 12 weeks

	0 week					12-week				
No-carrageenan diet	Oral disposition index[a]	HOMA-IR[b]	Matsuda Index[c]	QUICKI[d]	HOMA-2.2 %S[e]	Oral disposition index	HOMA-IR	Matsuda Index	QUICKI	HOMA-2.2 %S
1	0.019	8.84	1.14	0.281	263	0.619	0.45	6.34	0.443	263
2	0.036	2.99	2.05	0.324	67	0.017	1.61	3.82	0.355	109
3	0.068	4.55	1.90	0.306	38	0.159	1.93	3.67	0.346	95
4	0.049	2.78	1.95	0.328	74	0.091	1.00	4.1	0.384	212
5	0.033	0.76		0.401	200	0.244	0.71		0.407	229
6	0.035	3.52	3.25	0.317	49	0.169	2.77	2.3	0.328	659
7	0.069	5.15	2.5	0.301	38	2.429	0.32	8.82	0.472	264
8	0.039	2.54		0.332	68	0.289	0.83		0.396	196
Carrageenan-containing diet										
1	0.622	0.22	7.93	0.512	114	0.061	1.33	4.47	0.366	124
2	0.260	0.85	5.12	0.394	201	0.105	0.92	8.08	0.389	177
3	0.028	7.31	1.31	0.288	27	0.025	4.10	2.17	0.311	54
4	0.155	3.11	3.05	0.323	58	0.192	1.90	4.74	0.346	102
5	0.033	8.40	1.73	0.283	20	0.152	6.63	2.37	0.292	24

[a] DIo= Oral disposition index = [ΔInsulin (0-30 minutes) / ΔGlucose (0-30 minutes)] / fasting insulin [40]

[b] HOMA-IR = glucose x insulin / 405

[c] Matsuda index - calculated from Web calculator: http://mmatsuda.diabetes-smc.jp/MIndex.html

[d] QUICKI = 1 / [log(fasting insulin) + log (fasting glucose)]

[e] HOMA2.2 calculated from Web calculator: https://www.dtu.ox.ac.uk/homacalculator/download.php
